# Residence Time, Water Contact, and Age-driven *Schistosoma mansoni* Infection in Hotspot Communities in Uganda

**DOI:** 10.4269/ajtmh.21-0391

**Published:** 2021-10-18

**Authors:** Arinaitwe Moses, Moses Adriko, Brian Kibwika, Edridah M. Tukahebwa, Christina L. Faust, Poppy H. L. Lamberton

**Affiliations:** ^1^ Vector Borne and Neglected Tropical Diseases Control Division, Ministry of Health, Kampala, Uganda;; ^2^ Cavendish University Uganda, Kampala, Uganda;; ^3^ Institute for Biodiversity, Animal Health and Comparative Medicine and Wellcome Centre for Integrative Parasitology, University of Glasgow, Glasgow, United Kingdom

## Abstract

Schistosomiasis is the second most important parasitic infection after malaria in terms of its socioeconomic impact and is endemic in 78 countries. It affects more than 240 million people worldwide, with 90% of cases occurring in sub-Saharan Africa. In Uganda, *Schistosoma mansoni* is the most common species, with more than seven million people infected and 17 million living at risk despite mass drug administration (MDA) of praziquantel initiated more than 16 years ago. There has been a shift in the WHO schistosomiasis goals from controlling morbidity to elimination as a public health problem. Understanding the drivers of infection in persistent transmission hotspots despite ongoing control interventions is paramount. We conducted a cross-sectional epidemiological study of 381 individuals in Bugoto community, Mayuge district, Eastern Uganda, along with a structured survey to ascertain drivers of *S. mansoni* infection. Bugoto has had community-wide MDA since 2004. We detected a *S. mansoni* prevalence of 52% across the whole community and a prevalence of 71% in school-age children. This qualifies Bugoto as a highly endemic community according to WHO guidelines. Using a multivariate logistic regression, we found that *S. mansoni* infection was best explained by age group, longer residence times, and any daily contact with lake water. *Schistosoma mansoni* infection remains a large burden across this community. This study identifies opportunities for interventions that reduce lake water contact, expand treatment eligibility to all at risk, and improve MDA coverage for long-term residents in these settings to control schistosomiasis in persistent transmission hotspots.

## INTRODUCTION

Schistosomiasis, which is the second most important parasitic infection after malaria in terms of its socioeconomic impact, is responsible for the loss of an estimated 4.5 million disability-adjusted life years (DALYs) worldwide.^1^ Schistosomiasis, including both intestinal and urinary forms of the disease, occurs in 78 countries across the globe. An estimated 240 million people are infected, with more than 779 million living at risk globally.^2^ The majority of those infected and those at risk for infection live in low-income countries,^3^ and approximately 80% of the morbidity occurs in impoverished communities and households in sub-Saharan Africa. Within Uganda, 91 of the 134 districts are endemic for intestinal schistosomiasis caused by *Schistosoma mansoni*, and the eastern region, especially along Lake Victoria, has one of the highest *S. mansoni* burdens worldwide.^4^^–^^7^ *Schistosoma haematobium* is only endemic in the five districts of the Lango region in northern Uganda.^8^

Schistosomiasis is considered a highly focal disease.^9^ High endemicity communities, defined by the WHO as having a prevalence of more than 50% in school-age children (SAC), can be found immediately adjacent to communities with low endemicity (<10% SAC).^10^ This focality is partly attributable to the presence of freshwater environments, suitable intermediate host snails, and inadequate sanitation. *Schistosoma mansoni* ova in infected human feces hatch into miracidia when in contact with freshwater. Miracidia undergo asexual reproduction and several developmental stages in *Biomphalaria* snails; they are shed into the water as an infective-stage cercariae.^8^ Humans become infected with *S. mansoni* when they contact water harboring these free-swimming cercariae.

Research has investigated both individual and community risk factors that influence schistosomiasis infection intensity and prevalence. Ecological features, including snail habitat and water conditions, are correlated with the presence and prevalence of schistosomiasis at the community level.^11^ Behavioral factors have been shown to influence individual risk, particularly swimming, bathing, and washing clothes in open water sources.^12^^,^^13^ Hygiene behaviors, such as open defecation, can lead to high community levels of infection with the disease, although this behavior is not directly linked to the individual’s risk for infection.^14^ Certain occupational activities, such as fishing and agricultural practices that involve irrigation and rice paddy growing, increase risk for infection.^15^ In addition, socioeconomic levels at both the family level and individual level influence schistosomiasis infection risk, as reported by Muhumuza et al.^16^ in Uganda.

Within Uganda, the national Bilharzia and Worm Control Program aimed to control schistosomiasis with the goal of elimination of the disease as a public health problem by 2020.^17^ However, as in most other countries, this was not accomplished. The WHO 2021–2030 neglected tropical disease roadmap now aims for 100% of endemic countries to have reached this goal by 2030.^20^ Mass drug administration (MDA) of praziquantel, health education, access to clean and safe water, and sanitation improvements are strategies recommended by the WHO to control the disease. As in many African countries, MDA is the mainstay of control in Uganda. This strategy uses WHO guidelines for treatment coverage and aims for 75% coverage in highly endemic communities (where the prevalence in SAC is > 50%) for all members 5 years of age and older.^18^ Although the control program has successfully reduced the burden of the disease, particularly when it was first intitiated,^19^ reduced efficacy of praziquantel,^6^ transmission bouncebacks,^20^ high reinfection rates,^21^ and low treatment coverage^25^ are real threats to the continued progress in Uganda and elsewhere. According to cluster randomized trials performed in other countries through a program called SCORE (Schistosomiasis Consortium for Operational Research and Evaluation), particular villages have had persistent high intensity and/or prevalence despite repeated MDA and are referred to as persistent hotspots.^22^ Bugoto community, like many others in Uganda, where our study was performed, has maintained high prevalence despite more than 14 years of MDA and remains a highly endemic community according to WHO endemicity categorization.

With the recent shift in focus by the WHO from morbidity control to elimination as a public health problem,^23^ there is a renewed need to understand drivers of disease transmission in communities that are not responding to standard control interventions. Understanding such drivers would aid disease control through the reorientation of interventions, the development of novel individual-focused and community-focused interventions, and informing program managers of alternative options for control. Schistosomiasis infection can be acquired in environments where infectious vectors are found; therefore, the infection risk is dependent on not only individual factors but also contributions to onward transmission by other members in the community at large.^24^ Lack of access to adequate sanitation facilities can lead to individuals defecating in the environment, thus increasing the risk and transmission of schistosomiasis to others. The main objectives of this study were to improve our understanding of the drivers of infection among individuals as well as quantifying risk factors in a persistent transmission hotspot in Eastern Uganda that has undergone praziquantel MDA since 2003.

## METHODS AND PROCEDURES

### Study area.

The study was conducted in Bugoto community, which consists of two villages, Bugoto A and Bugoto B, on the shores of Lake Victoria in Bukabooli sub-county, Mayuge District, Eastern Uganda. Bugoto community is served by a level-two government health center, one government primary school, and a few smaller private primary schools. A household census conducted in February 2017 in Bugoto A and Bugoto B demonstrated that these are predominately small-scale agriculture and fishing communities, respectively.^25^ These villages are representative of small rural communities along water bodies.^26^ National school-based MDA of praziquantel began in 2003; in 2004, it was scaled-up to cover the whole community on an annual basis. In 2019, 1 year after data collection for this study, community-wide MDA was scaled-up again to biannual treatment. Bugoto community was chosen for this study because it remains highly endemic for intestinal schistosomiasis^6^^,^^7^^,^^27^ and had undergone annual community-wide MDA of praziquantel for more than 14 years at the time when the study was undertaken, which is the maximum time for any community in Uganda. Despite receiving community-wide MDA, transmission and infection remain high; therefore, additional interventions are needed in this area and in similar areas.

### Survey design and data collection.

**Figure 1.**
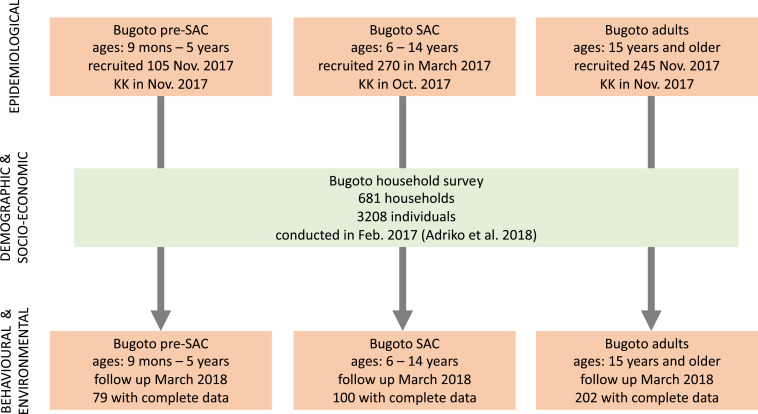
Recruitment and data collection flow. Timing and sample size of recruited individuals during each data collection event. This figure appears in color at www.ajtmh.org.

The disease burden across Bugoto community was estimated at 50% according to previous studies. Using a sampling error of 0.5% at a 95% CI and z-score of 1.96,^28^ the minimum sample size required to differentiate the infection prevalence and lead to understanding the drivers of acquiring schistosomiasis infection within this community was calculated as 384 individuals. All individual members of the community were first registered during a community-wide household survey in February 2017.^25^ Epidemiological cohorts were recruited in March 2017 (SAC 6–14 years of age) and November 2017 (preschool-age children [PSAC] 9 months–5 years and individuals older than 14 years) (Figure 1). Individuals were randomly selected from the community register to represent equal sex and age ratios. The Uganda population and housing report^26^ offers estimates of the age structure in 5-year increments (approximating each age group); according to that report, 19.5% are PSAC (defined as younger than 5 years; however, PSAC in Uganda are commonly younger than 6 years), 34% are SAC (age 5–14.9 years), and 46.3% are “adults” who are 15 years or older. Individuals were proportionally sampled from these three age groups but defined as slightly different age categories (Figure 1) as follows: 75 PSAC, 131 SAC, and 178 adults. Children younger than 9 months were not sampled because of ethical constraints associated with treatment. Follow-up of the participants to obtain behavioral and knowledge data was conducted in March 2018. The subset for these behavioral and knowledge surveys were randomly selected from the individuals who had previously completed the household survey and provided samples for the epidemiological cross-sectional survey in March 2017 for SAC and in November 2017 for PSAC and adults (Figure 1).

At the time of the initial recruitment, and at the time of each subsequent follow-up survey, study procedures were explained to the participants; full ethical consent and assent were required for participation. Children younger than 15 years were consented by their parents/guardians, and children 8 years and older also provided assent in addition to parental/guardian consent. A semi-structured questionnaire was administered by Vector Control Officers to all consenting individuals. Parents, guardians, and/or older siblings completed questionnaires on behalf of children younger than 5 years. Questions were designed to determine specific individual demographic, behavioral, environmental, and socioeconomic characteristics related to the acquisition of intestinal schistosomiasis (Table 1). Demographic variables collected included age, sex, and religion. Environmental variables included the number of years an individual has been a resident of the community and the approximate distance of the house from the lake shoreline.

**Table 1:** Variables collected during the survey

Demographic data
Sex
Religion
Age (in years) Age group (PSAC, SAC, adult)
Environmental variables
Contact with lake (yes/no)
Zone where lake contact occurs
Household distance from the lake
Number of years of residence in the community
Behavioral variables
Praziquantel MDA compliance ever
Praziquantel MDA compliance last year
Frequency of lake water contact Duration of lake water contact Water contact activities
Latrine usage at work and at home
Socioeconomic variables
Latrine ownership
Socioeconomic status
Knowledge of schistosomiasis
Occupation

MDA = mass drug administration; PSAC = preschool-age children; SAC = school-age children.

Contracting schistosomiasis involves contact with water with intermediate vector snails that are shedding larval cercariae; however, not all water contact sites have an equal risk. Mapping out frequently visited contact sites along the lake shoreline for this community and differentiating risk (through infection status) would help identify transmission sites where control interventions could be targeted. Based on 15 years of working in this community, we divided water contact sites by the types of activities conducted there. These were also partitioned into the following different ecological zones: zone 1, swampy shoreline where some community members go to get water as well as grow rice; zone 2, rocky far end of the community extending into lake and 10 m from the shoreline that is used by community members for fishing and transit to islands; and zone 3, open lake shoreline stretching more than 300 m but not exceeding 10 m from the shoreline, with some floating vegetation, that is used mostly for swimming, gathering household water, and transit (Supplemental Figure S1).

The reported behavioral variables in this survey focused on lake water contact and MDA compliance. Reported lake water contact (frequency, duration, and activities) was recorded. Praziquantel MDA compliance was assessed based on individual recall both within the previous MDA campaign (July 2017) and ever during their lifetime. Survey questions also collected data regarding latrine usage at home and work.

Intestinal schistosomiasis is a neglected tropical disease that is strongly associated with low socioeconomic development. To ascertain the socioeconomic status of the household, we used an established method to assign values to different construction material within households^29^ (house floors: mud = 1, cement = 2, tiles = 3; walls: mud = 1, bricks = 2, bricks and plaster = 3; and roofing: grass-thatched = 1; papyrus = 2; iron sheets = 3). In addition, we collected information regarding latrine ownership, water source, and level of education. We divided latrine ownership into three categories: no latrine = 0; shared latrine = 2; or private latrine = 3. We scored water sources as follows: lake = 1; borehole = 2; and piped water = 3. The level of education was scored as follows: no education = 0; primary level = 2; and secondary level or more = 3. Then, we used these scores to create the socioeconomic status of the household (household head and family members) based on the work by Filmer and Pritchett.^30^

Finally, we asked participants questions about modes of transmission, effects of infection on individuals, and types of control methods to ascertain awareness and knowledge of schistosomiasis (see Supplemental Information for full questionnaires).

### Epidemiological status.

We asked each participant to provide stool samples in October 2017 (SAC) and November 2017 (PSAC and adults). We processed samples using the Kato-Katz thick smear technique.^28^ To improve the accuracy of infection status for low-intensity infections, three consecutive daily stool samples were collected from each participant. These samples were prepared on duplicate slides and read by highly trained Vector Control Officers from the Ministry of Health under a compound microscope (×10 objective). Before being approved to read slides, Vector Control Officers must complete a course that allows them to learn how to evaluate the sensitivity and specificity of Kato-Katz parasitological readings. We used to recheck 25% of slides, but errors were rare and did not significantly affect the prevalence or intensity measures; therefore, we halted this practice to conserve resources. We did not conduct parasitological surveys of *Schistosoma haematobium* because it is not endemic in this community or district, despite being repeatedly tested for over the years, including by the authors. We defined a positive schistosomiasis status as the presence of at least one *S. mansoni* ova on any Kato-Katz slide.

### Data analysis.

We double-entered parasitological and survey data into Excel and cleaned the data. We created univariate logistic regressions to evaluate which demographic, environmental, behavioral, and socioeconomic variables were predictive variables of the infection status. Then, we performed multivariate logistic regression with all significant univariate variables to determine the most important factors influencing the risk of acquiring intestinal schistosomiasis, as measured by presence of eggs in stool. Model selection was performed in a stepwise manner, and the best-fit model was selected using the Akaike information criterion.

### Ethical consideration.

We conducted this study within the framework of the ongoing Bilharzia and Worm Control Program of Ministry of Health and the European Research Council funded SCHISTO_PERSIST 688088 project (to PHLL). Approvals were obtained from the Vector Control Division Research Ethics Committee (VCDREC/062), Uganda National Council for Science and Technology (UNCST-HS 2193), and Veterinary and Life Science Research Ethics Committee of the University of Glasgow College of Medicine (MVLS 200160068). We also obtained permission from Mayuge district local government administration. We obtained signed/thumbprint informed consent from all adults and the parents or guardians of SAC and PSAC. We obtained assent from all SAC. We treated all SAC and adults diagnosed as schistosomiasis-positive with 40 mg/kg praziquantel. Positive PSAC (9 months–5 years of age) were treated with 60 mg/kg. Participants could withdraw themselves or their children from the study at any time without it affecting their access to praziquantel treatment during this study or through community MDA.

## RESULTS

After recruitment was performed and informed ethical consent was obtained, a total of 381 individuals participated in the study; of these, 50.1% were male (Figure 1, Table 2). A higher proportion of males was infected with *S. mansoni* (55.5%), although the difference was not significant (Table 3).

**Table 2:** Characteristics of study participants and infection status (*N* = 381)

Group	Enrolled, n (%)	*S. mansoni-*positive, n (%)
Age group		
9 months–4.9 years	79 (20.7%)	31 (39.2%)
5–14.9 years	100 (26.2%)	71 (71.0%)
15 years or older	202 (53.0%)	96 (47.5%)
Sex		
Male	191 (50.1%)	106 (55.5%)
Female	190 (49.9%)	92 (48.4%)
Religion		
Islamic	170 (44.6%)	84 (49.4%)
Catholic	76 (19.9%)	48 (63.2%)
Protestant	103 (27.0%)	49 (47.6%)
Other*	32 (8.4%)	17 (53.1%)

* Other religious faith included Pentecostal and traditional sect.

**Table 3:** Univariate analysis results

			Significance		
Category	Variables	OR	*P* < 0.1; *P < 0.05	2.5% CI	97.5% CI
Demographics					
Sex	Female	0.94		0.71	1.25
	Male	1.33		0.89	1.99
Religion	Christian (intercept)	1.18		0.90	1.54
	Muslim	0.83		0.55	1.25
Age (years)	(Intercept)	1.39	*	1.01	1.93
	Age	0.99	*	0.98	1.00
Age group (PSAC, SAC, adult)	PSAC (intercept)	0.60		0.38	0.95
	SAC	4.06	*	2.20	7.65
	Adults	1.48		0.87	2.56
Environmental					
Zone where lake contact occurs	Bushy	1.50		0.25	11.39
	Landing of the health facility	0.93		0.12	5.71
	Rocky open waters	0.52		0.06	3.70
Household distance from the lake		0.82		0.57	1.17
	Distance from the lake	1.51	*	0.98	2.34
Number of years of residence in the community (group)	< 5 years	0.63	*	0.43	0.91
	5–9 years	2.66	*	1.57	4.53
	≥ 10 years	1.89	*	1.16	3.11
Number of years of residence in the community (proportion)	(Intercept)	0.50	*	0.31	0.80
	Residence	2.97	*	1.64	5.46
Village	A	1.23		0.96	1.57
	B	0.66		0.43	1.03
Behavior					
PZQ MDA compliance ever	(Intercept)	0.83		0.59	1.17
	PZQ ever	1.14		0.72	1.82
PZQ compliance last year	(Intercept)	0.82		0.64	1.05
	PZQ last year	2.17	*	1.42	3.35
Contact with lake (yes/no)	(Intercept)	0.33	*	0.18	0.57
	Lake visit	4.04	*	2.25	7.63
Frequency of lake water contact	None	1.51	*	1.03	2.24
	A few times per month	1.00		0.60	1.67
	A few times per week	0.73		0.35	1.52
	Once per day	0.23	*	0.11	0.44
	Twice per day	0.41		0.12	1.32
	Three times per day	0.51		0.17	1.48
Duration of lake water contact	None	0.33	*	0.18	0.57
	< 5	3.54	*	1.68	7.69
	5–15 min	4.01	*	2.15	7.83
	16–30 min	4.73	*	2.27	10.23
	> 30 min	3.90	*	1.45	10.88
Latrine usage at work	Never	0.91		0.59	1.39
	Sometimes	0.86		0.47	1.55
	Always	1.48		0.89	2.45
Latrine usage at home	Never	1.2		0.36	4.16
	Sometimes	0.99		0.27	3.48
	Always	0.86		0.24	2.92
Socioeconomic status					
Latrine ownership	None	1.00		0.12	8.33
	Private	0.98		0.12	8.30
	Shared	1.19		0.14	10.09
Socioeconomic status	Low	1.01		0.80	1.29
	Medium	1.21		0.77	1.90
	High	2,087,258.00		0.00	NA
Knowledge of schistosomiasis	None	0.80		0.60	1.07
	Low	1.76		0.89	3.53
	Medium	1.80	*	0.93	3.55
	High	1.78	*	1.11	2.88
Education	None	0.76		0.52	1.12
	Started primary school	1.79		1.11	2.91
	Finished primary school	0.40		0.11	1.23
	Started secondary school	1.55		0.82	2.97
	Finished secondary school	7,549,132.00		0.00	NA
	Secondary school or more	0.66		0.03	7.05

MDA = mass drug administration; PSAC = preschool-age children; PZQ = praziquantel; SAC = school-age children.

**Table 4:** Multivariate analysis

	Adjusted OR	2.50%	97.50%
PSAC	0.7	0.05	1.94
SAC	0.0008	0.00005	0.02
Proportion of residence time	1.52	0.62	3.62
Duration of water contact			
< 5 min	2.65	1.12	6.41
5–15 min	3.2	1.58	6.8
16–30 min	3.85	1.67	9.22
> 30 min	2.59	0.84	8.14
Age group			
PSAC: Residence proportion	1.06	0.36	1.8
SAC: Residence proportion	6.73	226	43.53

PSAC = preschool-age children; SAC = school-age children.

Of the 381 participants, the age structure mirrored the population average, with 20.7% PSAC (79/381), 26.2% SAC (100/381), and 53.0% adults (202/381). The prevalence of *S. mansoni* in SAC was 71.0%, thus confirming Bugoto as a highly endemic community as defined by the WHO. Furthermore, more than one-third (39.2%) of PSAC had detectable *S. mansoni* egg infection (Table 2). Across the whole community, the overall prevalence was 52.0%.

The majority of participants (50.6%) had access to shared latrines, and a slightly lower percentage (47.3%) had a private latrine, whereas only 1.0% reported no latrine access. Up to 22.6% of participants reported that they did not use latrines at all at work, 23.4% sometimes used them at work, and 3.2% reported not using latrines at all. A slightly larger proportion of the participants reported using latrines at home, although this difference was not significant.

### Individual risk for infection with *S. mansoni.*

Age in years was not a significant predictor of the *S. mansoni* infection status; however, age group (PSAC, SAC, adult) was. Compared with the *S. mansoni*-negative infection status, according to a univariate analysis, SAC were 4.06-times more likely to be infected with *S. mansoni* than PSAC (odds ratio [OR]: 0.60; 95% confidence interval [CI]: 0.38–0.95) or adults (OR: 1.48; 95% CI: 0.87–2.56) (Table 3). The majority of study participants (83.2%) reportedly visited the lake. Some only visited the lake a few times a month (3.4%) whilst others visited it up to three times per day (28.3%). PSAC were less likely to visit the lake (53.2%) than SAC (95%) and adults (89.1%). Most participants reported their main lake water contact activity was gathering water (27.6%); other activities included swimming and fishing. The duration of water contact was significantly associated with the *S. mansoni* infection status. Individuals who reported never contacting the water had only a third of the chance of being infected compared to those who contacted the water at all, even if only for less than 5 minutes. Of the individuals who reported not visiting the lake, 25% were infected with *S. mansoni*; of those 25%, the majority were PSAC (median age, 2.74 years). The distance from an individual’s home to the lake was not significant for predicting *S. mansoni* infection, nor was the zone where individuals contact lake water. Residing in Bugoto community for 5 or more years was positively associated with the *S. mansoni* infection risk (OR: 2.66; 95% CI: 1.57–4.53) compared with residents who had lived there fewer than 5 years (OR: 0.63; 95% CI: 0.43–0.91).

We observed a knowledge gap surrounding schistosomiasis disease, transmission, and control among participants. Fewer than half were able to correctly identify modes of transmission (44.6%), the impact of infections (33.3%), or control methods (43.8%). However, knowledge of all three was associated with a higher risk of infection with *S. mansoni* (OR: 1.78; 95% CI: 1.11–2.88).

Praziquantel MDA compliance rates of the community were below the WHO targets: only 36.2% of eligible individuals reported receiving treatment during the past year. Praziquantel treatment during the past year was positively associated with *S. mansoni* infection (OR: 2.17; 95% CI: 1.42–3.35). Furthermore, 62.5% of the eligible participants reported receiving praziquantel at least once during their lifetime, although this was not predictive of the current *S. mansoni* infection.

Although several factors were important predictors of *S. mansoni* infection when examined in isolation, only a few of these were important when controlling for all possible combinations using a multivariate model. The best-fit model indicated that age group, proportion of lifetime residence, and duration of water contact were all significant predictors of *S. mansoni* infection (reported as adjusted OR [aOR]). This best-fit model indicated a significant interaction between age group and residence time, meaning that the impact on age is dependent on the proportion of the lifetime spent locally (Table 4). SAC who had lived a small proportion (< 10%) of their lifetime in Bugoto were at low risk for infection (aOR: 1.06; CI: 0.36–1.8). However, living an entire lifetime in Bugoto was associated with a 6725-times increase in the infection risk (aOR: 6725; 95% CI: 226–43525).

It was observed that any duration of lake water contact, regardless of frequency, was associated with an increased *S. mansoni* infection risk. Although praziquantel MDA compliance was significantly negatively associated with infection risk when analyzed using univariate logistic regression, it was not present in the best-fit model. Additionally, socioeconomic variables and knowledge of schistosomiasis were not included in the final best-fit model of individual risk for acquiring *S. mansoni* infection because they were no longer significant.

## DISCUSSION

Schistosomiasis is a complex disease, and its infection risk is closely linked with individual and community behaviors, the environment, and control interventions. Our study focused on a persistent transmission hotspot to identify key drivers of infection and quantify risk factors to target the existing control interventions. We demonstrated that *S. mansoni* remains a public health problem in Bugoto community (71% prevalence for SAC) despite more than 14 rounds of praziquantel MDA. The community has several high-risk factors, including inadequate sanitation, high-frequency water contact, and low treatment coverage,^25^ resulting in a high infection rate and endemicity.^20^ Despite these many community-wide factors, we found that the *S. mansoni* infection risk was best predicted by age group, duration of lake water contact, and residence time within the community, thereby suggesting characteristics to select for when designing and targeting interventions. Results from communities like this that have undergone several rounds of MDA demonstrate the current inadequacy of solely using MDA for schistosomiasis control and elimination in persistent transmission hotspots.

During our study, lake water contact for as little as 5 minutes per day was significantly associated with the risk for *S. mansoni* infection, suggesting a high force of transmission in this community. Although the duration of water contact was significant, the frequency of water contact was not. People who never contact lake water have already been controlled for in the significant zero-minute duration of water contact group; therefore, anyone who contacts lake water, even for a few minutes, is at risk. It does not matter how often they have contact with water because they have likely already been exposed and have become infected. By living in a community that almost solely depends on lake water for their water source, the high frequency of lake water contact does not appear to substantially increase the risk of being infected because any contact could have resulted in infection. This heavy dependence is characteristic of many landing sites on large water bodies in sub-Saharan Africa that lack clean water for basic activities.^31^^,^^32^ Several activities like gathering water,^33^ swimming, fishing, fish mongering, and transit to different islands expose residents to lake water contact.^34^ The majority of these activities are routine for most residents and usually involve approximately 5 minutes or more of water submersion. Therefore, because contact with the lake water is associated with infection, simple measures that enable people to collect water without entering it (such as using jetties) or telling people to wash their clothes while standing on the shore rather than in the water could be effective. Certain occupations require water access; therefore, additional interventions might be required, such as the use of wellies for farmers and boat jetties and dry landing sites for fishermen. With an increased cercaria-infested lake water contact, we expect that the intensities of infection will increase; however, the size of the study population did not allow us to test this hypothesis robustly.

During this study, the majority of lake water contact involved gathering water. Other water sources such as boreholes are available in these communities. However, there is only one in Bugoto A and one in Bugoto B; they both frequently break, have long queues during the dry season, and are only used for safe drinking water, if at all. A community piped water system broke several years ago, but even when it was working, water was at a cost that was prohibitive to most community members. Therefore, lake water contact will remain unavoidable until a sustainable, popular, and affordable alternative is available. People need water to live, and the lake water is accessible and free, even if it is unsafe. There is an urgent need to make taps, boreholes, and/or wells more affordable, accessible, and safe so that people can be persuaded to use them (e.g., well water often tastes or lathers differently than lake water and can be less popular^35^). Education could help to ensure that people know that water that does not lather still washes the body and clothes well; however, personal taste preferences would likely be more difficult to change.^36^

We found that longer residence time in the community significantly increased the risk of acquiring *S. mansoni* infections. The intensity and morbidity associated with schistosomiasis are attributable to several exposures, with new additive infections acquired over time.^4^ The importance of residence time has been reported by others to be strongly associated with schistosomiasis infection.^4^^,^^37^ Targeting residents who have stayed in such a high-transmission hotspot community for more than 10% of their lifetime with MDA should help address individual morbidity and reduce schistosomiasis transmission. This could prove to be cost-effective in terms of successfully targeting infected individuals and potential transmitters during the process of elimination.

An informed individual is expected to perform measures that would limit the risk of *S. mansoni* infection. We report that knowledge of schistosomiasis was actually positively associated with infection. This is the opposite of what we would expect. However, greater knowledge with reduced infections could only be expected if people have the option to change their behavior. It may be that education and community sensitization are working and are reaching people at high risk, thus increasing their knowledge about the disease, transmission, and symptoms; however, the absence of alternative options,^38^ such as safe water sources, limits their abilities to protect themselves because they still need to use lake water.

The reported low MDA compliance/use among community members may be attributed to the fatigue of the volunteer drug distributors, as reported by Knopp et al.,^39^ not being offered treatment,^25^ individuals believing that they are not infected,^25^ conflicting agricultural activities,^40^ and the bitter taste of praziquantel.^40^ A positive association of previous MDA compliance with infection could be explained in the same way as we attributed the effects of knowledge, with those who are exposed being the ones who seek treatment and fear the consequences of the disease but have limited options to change their behavior after treatment and, therefore, are rapidly reinfected. The absence of MDA treatment and knowledge from the best-fit model could be attributable to the inability to change behaviors with treatment and knowledge; therefore, the actual exposure risk factors become the more important factors that drive the infection status. Alternatively, targeting treatments to SAC who are assumed to be at high risk and achieving high treatment coverage in schools and to PSAC who are not treated may mask the effects in the multivariant model. One limitation of this study was that the source of data used to ascertain praziquantel MDA compliance was interview answers, which are prone to recall bias. This recall bias, however, was minimized by showing participants tablets and describing the bitter taste. Furthermore, the total proportion of people who reported using praziquantel was similar to that reported by another study performed in the same village in 2017.^25^

A high proportion of community members (22.6%) reported not always using latrines at work and 3.2% reported that they did not always use latrines at home. This could be attributed to a general lack of physical latrines or access to them at work, especially for fishermen and farmers, individuals being too far from home when a latrine was needed, or individuals not wanting to use latrines. The lack of a latrine door is not uncommon; therefore, latrines lack privacy. Additionally, latrines often lack cleanliness, particularly communal latrines that are common on landing sites, making them uninviting. Furthermore, when latrines are locked, they are impossible to use. All of these contribute to lower usage even when they are present. This inconsistency in latrine use behavior often leads to open defecation that contaminates the environment with feces laden with *Schistosoma* ova.^41^ Therefore, with few alternatives to lake water contact, contracting *S. mansoni* is likely to occur in such communities.^42^^,^^43^

A critical observation for program managers and policy-makers is the high burden of potent *S. mansoni* eggs (39.2%) in children younger than 5 years (PSAC). Our findings support other studies performed in the same community that reported a high disease burden for children younger than 5 years^44^ and other studies across sub-Saharan Africa.^45^^–^^47^ Several scenarios explain the infection status of PSAC. PSAC contract the disease both actively and passively. Approximately half of the PSAC were reported to have visited the lake during the survey compared to 95% of SAC or approximately 90% of adults; however, this might be an underestimation and is based only on the answers provided by specific respondents. PSAC become actively infected by visiting the lake with their mothers/guardians or older siblings to collect water for domestic use, bathing, washing clothes by the lake, and swimming.^48^ PSAC are not always with the main parent/guardian/survey respondent, and the siblings may take the PSAC to the lake without mentioning it to the specific survey respondents. Additionally, there were PSAC infected with *S. mansoni* whose parents/guardians reported that they did not visit the lake at all, indicating that the PSAC went without them, the lake visit information was not fully or accurately recorded, or the infection was passive. PSAC may passively contact cercaria-infested water when they are bathed in water collected from the lake with viable cercariae. One potential way to reduce this type of infection is to improve education among parents and telling them to avoid using lake water for 24 to 48 hours after collection so that the viable cercariae die before use or to use other immediate treatment options such as chlorination or filtration to kill the viable cercariae.

Most MDA has focused on SAC because of their vulnerability and the ease of access for treatments. However, in hotspot communities, young adults, in particular, have low praziquantel MDA compliance rates,^25^^,^^49^ high disease rates, practice open defecation, and could have an important role in the rapid infection and reinfection rates of SAC and PSAC.^33^ Although other studies have also observed high burdens in all ages,^41^^,^^50^ PSAC are not targeted by the control programs, and there is no approved pediatric formulation for this group. This maintains the status quo of health inequity, with PSAC heavily infected and showing morbidity^51^^–^^53^ before they even reach school age. Whatever the cause of these infections in PSAC, it is vital that they are treated. Based on the WHO grade levels of schistosomiasis and praziquantel MDA, this group (PSAC) deserves MDA.

## CONCLUSION

*S. mansoni* is a public health problem in Bugoto community despite more than 14 years of MDA. Improving MDA coverage with targeted treatment strategies to at-risk residents who have lived more than 10% of their lifetime in the community would help control schistosomiasis and, therefore, help communities move toward the elimination of* S. mansoni* as a public health problem. Because much of the population visits the lake for water collection, the provision of alternative water sources, such as boreholes and piped water, that are easily accessible and affordable would limit lake water contact and the associated risk. Building lake barriers such as jetties so that people can collect water without water contact, building platforms for washing clothes in the absence of alternative water sources, encouraging wellie use by farmers, encouraging the use of boats to reach dry land, and improving education methods surrounding delayed water contact after collection for PSAC bathing are suggested.

## Supplemental Material


Supplemental materials
