# Supplementary material for: Residence Time, Water Contact, and Age-driven Schistosoma mansoni Infection in Hotspot Communities in Uganda
Source: Am J Trop Med Hyg. 2021 Oct 18;105(6):1772–81. doi: 10.4269/ajtmh.21-0391 (PMC8641335; doi:10.4269/ajtmh.21-0391)
Supplement: Supplementary file 1 [file tpmd210391.SD1.pdf]

Supplementary Information File 1for:

Residence time, water contact and age-driven *Schistosoma mansoni* infection in persistent transmission hotspot communities in Uganda.

Arinaitwe Moses[1 2], Adriko Moses[1], Kibwika Brian[2], Edridah M. Tukahebwa[1], Christina L. Faust[3+] and Poppy H. L. Lamberton[3+]

[1]Vector Borne and Neglected Tropical Diseases Control Division, Ministry of Health, Po Box 1661 Kampala, Uganda. [2] Cavendish University Uganda, Po Box 33145 Kampala, Uganda. [3] Institute for Biodiversity, Animal Health and Comparative Medicine & Wellcome Centre for Integrative Parasitology, University of Glasgow, Glasgow, UK. [+]Joint Senior Authors.

SI 1 Figure 1: Map of Bugoto community lakeshore zones

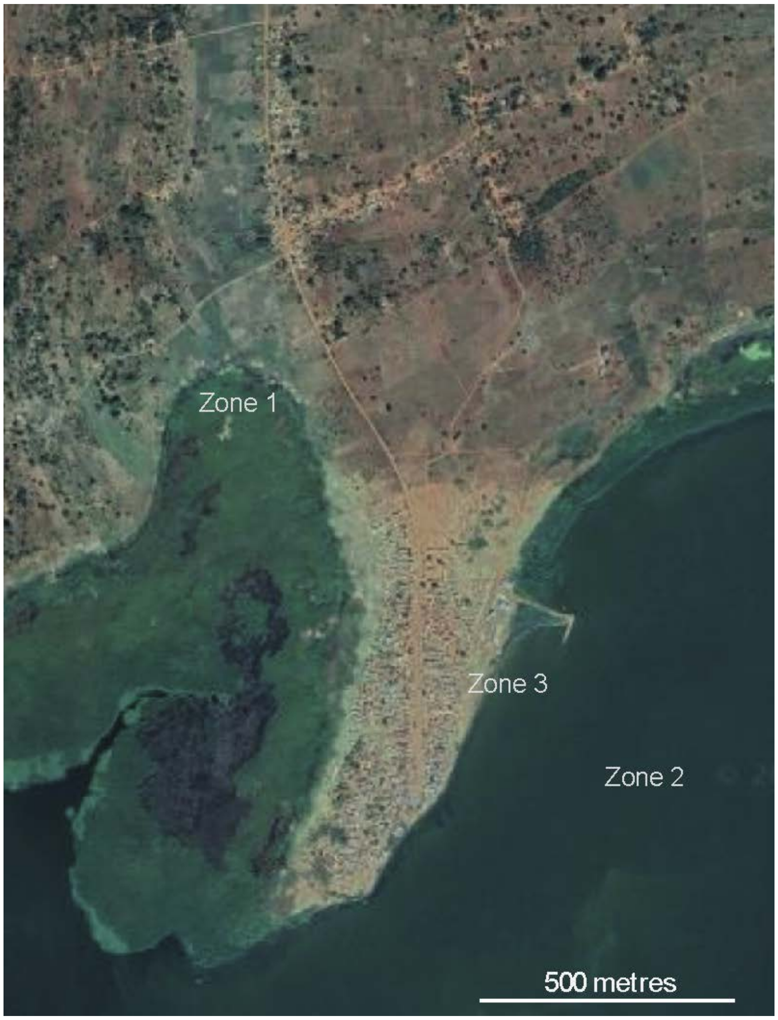

SI 1 Details of questionnaire administered:

PART 1 (previously published in full, in Supplementary Material, Supplementary File 1, of https://www.mdpi.com/2414-6366/3/4/111)

1. Demographics……
2. Age…………
3. Sex………………
4. Religion……………..
5. Occupation…………………………………………………………………
6. How long have you lived in this community?
7. What is the main source of water for household activities? Lake/borehole/pipe water/swamp.
8. Approximately how far is it to the main source of water (in meters) ……….
9. Did you take bilharzia drugs in the last year? Yes/No
10. Have you ever taken bilharzia drugs? Yes/No

PART 2

A. Do you visit the lake?

Yes/No

B. Why do you visit the lake? (Activities you do on the lake) Pick all that apply.

1) Fishing
2) Bathing
3) Washing motor bike
4) Collecting water for home use
5) Swimming/playing
6) Fish mongering
7) To carry people and their goods to/from the boats,
8) Others……………….

C. How often do you contact lake water from the lake?

1) X1 day
2) X2 day
3) X3 day
4) A few times a week
5) A few times a month
6) Never

Duration of lake water contact

D. How long are you in the water (with at least your feet or hands submerged) when you do contact water?

1) Under five minutes
2) 5-15 minutes
3) 16-30 minutes
4) 31+ minutes

E. Which area of the lake do you visit regularly?

Note the zones (see map) where they contact water (circle the most frequently used zone)

1) 1: Zone one with bushy shoreline
2) 2: Zone two consisted of the rocky and mid lake ten meters from the shoreline
3) 3: Zone three the open shoreline with some submerged and floating vegetation

F. Do you use a latrine always when at work/ school?

1) Yes
2) Sometimes

3) No

G. If not always, why?
1) Too far away
2) Not available in the work field/lake
3) Very dirty to be used
4) Others……………….

H. Do you use latrine always when at home?
1) Yes
2) Sometimes
3) No

I. If not always, why?
1) Too far from home
2) Not available
3) Not well lit at night
4) No doors
5) Very dirty
6) Others……………….

Wealth Index (if Head of Household)

J. Do you own any of the following?
1) Radio
2) Tv
3) Boda boda (Motocycle)
4) Landownership
5) Private latrine
6) Personal house
7) Bed
8) Solar
9) Boat

K. House
1) Floor: a) Mud b) Cement
2) Wall: a) Mud b) Brick c) Cement
3) Roof: a) Grass b) Iron sheet
4) Sleep: a) Floor b) Bed

L. Highest level of education attained
1) Never went to school
2) Started but have not completed primary level
3) Completed primary level
4) Started but have not completed ordinary secondary level
5) Completed ordinary secondary level
6) Started but have not completed advanced secondary level
7) Completed advanced secondary level

M. Have you ever heard of bilharzia disease?
1) Yes
2) No

N. How do you get bilharzia? (Tick all that apply)

1) Playing in water
2) Drinking dirty water
3) Swimming in lake
4) Fetching water
5) Others………………….
6) I don't know

O. How does Bilharzia affect your body? (Tick all that apply)

1) It leads to stomach pains
2) Bloody stools
3) General body weakness
4) Extended abdomen
5) Others......................
6) I don't know

P. How can somebody avoid contracting or stop transmitting bilharzia? (Tick all that apply)

1) Taking medicines
2) Avoiding lake water contact
3) Drinking boiled water
4) Using latrines all the times
5) Others…………………..
6) I don't know
